# Small molecule modulation of the large-conductance calcium-activated potassium channel suppresses salicylate-induced tinnitus in mice

**DOI:** 10.3389/fnins.2022.763855

**Published:** 2022-08-25

**Authors:** Luisa L. Scott, Andrea S. Lowe, Elliott J. Brecht, Luis Franco-Waite, Joseph P. Walton

**Affiliations:** ^1^Cognosetta Inc., Tampa, FL, United States; ^2^Department of Chemical and Biomedical Engineering, University of South Florida, Tampa, FL, United States; ^3^Global Center for Hearing and Speech Research, University of South Florida, Tampa, FL, United States; ^4^Department of Communication Sciences and Disorders, University of South Florida, Tampa, FL, United States

**Keywords:** tinnitus, auditory, potassium channel, BK channel, auditory midbrain

## Abstract

Tinnitus is the phantom perception of sound that has no external source. A neurological signature of tinnitus, and the frequently associated hyperacusis, is an imbalance between excitatory and inhibitory activity in the central auditory system (CAS), leading to dysregulated network excitability. The large conductance, calcium-activated potassium (BK) channel is a key player in pre- and post-synaptic excitability through its mediation of K^+^ currents. Changes in BK channel activity are associated with aberrant network activity in sensory regions of the CNS, raising the possibility that BK channel modulation could regulate activity associated with tinnitus and hyperacusis. To test whether BK channel openers are able to suppress biomarkers of drug-induced tinnitus and hyperacusis, the 1,3,4 oxadiazole BMS-191011 was given to young adult CBA mice that had been administered 250 mg/kg sodium salicylate (SS). Systemic treatment with BMS-191011 reduced behavioral manifestations of SS-induced tinnitus, but not hyperacusis, probed *via* the gap-in-noise startle response method. Systemic BMS-191011 treatment did not influence SS-induced increases in auditory brainstem response functions, but local application at the inferior colliculus did reverse SS-suppressed spontaneous activity, particularly in the frequency region of the tinnitus percept. Thus, action of BMS-191011 in the inferior colliculus may contribute to the reduction in behaviorally measured tinnitus. Together, these findings support the utility of BK channel openers in reducing central auditory processing changes associated with the formation of the tinnitus percept.

## Introduction

Subjective tinnitus—the phantom perception of sound that has no external or internal acoustic source—is an audiological and neurological disorder. Moderate to severe tinnitus affects 25 million Americans, and is highly prevalent among current and former Military Service members (Moller, 2011). There is currently no FDA-approved medication for tinnitus. Evidence from animal models and humans suggests that tinnitus can arise after peripheral injury and a resulting focal loss of cochlear sensitivity, although not all cases share this etiology. Loss of cochlear sensitivity is commonly caused by noise exposure, aging, or ototoxic drugs. Additionally, administration of the drug, sodium salicylate, reliably induces reversable peripheral hearing loss and tinnitus in both humans and animal models. Multiple modes of action support salicylate-induced changes in central auditory system (CAS) function. Direct peripheral and auditory nerve effects of salicylate administration that support tinnitus include raised cochlear thresholds and abnormal NMDA receptor activation in spiral ganglion neurons (Guitton et al., 2003; Puel and Guitton, 2007). A direct central effect also enhances sound-evoked hyperactivity in the CAS (Yang et al., 2007; Sun et al., 2009; Chen et al., 2013). This latter action is also linked to hyperacusis, an intolerance to loud sounds that is highly comorbid with tinnitus. While the entirety of salicylate effects are unlikely to be shared across all tinnitus etiologies, salicylate administration provides a highly uniform model for studying the modulation of tinnitus, increased central gain, and dysregulated activity in the CAS (Eggermont, 2013).

Following permanent cochlear injury or temporary loss of sensitivity, maladaptive plasticity in the CAS can alter spontaneous firing and neural synchrony in brainstem and cortical regions, increase central gain, and alter cortical networks (Jastreboff and Sasaki, 1986; Ochi and Eggermont, 1996; Syka and Rybalko, 2000; Basta and Ernst, 2004; Kaltenbach et al., 2005; Zhang et al., 2011; Chen et al., 2013; Shore et al., 2016). Though the precise relationships between these neurophysiological changes are not fully understood, aspects of this dysregulated neural and network activity interact to support the generation of tinnitus and hyperacusis (Salvi et al., 2000; Lanting et al., 2009; Norena, 2011; Radziwon et al., 2017). The large conductance, calcium-activated potassium (BK) channel has been a target of interest for other brain disorders characterized by an imbalance in neuronal and network excitability, like temporal lobe epilepsy, tonic-clonic seizures, alcohol withdrawal seizures and Fragile X syndrome (N’Gouemo, 2011; Deng and Klyachko, 2016; Scott et al., 2018). BK channels are composed of a pore forming α-subunit and an array of auxiliary subunits, with the β1, β4, and γ3 subunits most common in brain (Contet et al., 2016). The BK channel regulates neurotransmitter release, synaptic integration and action potential firing in the nervous system (Contet et al., 2016). Dual activation by intracellular calcium (Ca^2+^) and membrane depolarization allows the BK channel to generate K^+^ currents in response to strong cellular excitation. Expressed pre- and post-synaptically, these channels contribute to the regulation of neurotransmitter release, postsynaptic signal integration, action potential shape, and firing patterns (Contet et al., 2016). Both decreasing and increasing BK channel activation are associated with network hyperexcitability, likely due at least in part to the complex interaction and varied expression patterns of ion channels that shape circuit dynamics (Marder and Goaillard, 2006; Fakler and Adelman, 2008; Fransen and Tigerholm, 2010; Ly et al., 2011; Kimm et al., 2015). In sensory regions, downregulation of BK channel function is associated with enhanced dendritic excitability and action potential signaling, as well as suppressed sensory habituation (Deng et al., 2013; Typlt et al., 2013; Zaman et al., 2017). This raises the BK channel as a target of interest for modulating network excitability changes associated with tinnitus and hyperacusis.

Expression of the channel throughout the peripheral and central auditory system is well situated to impact the neurophysiological underpinnings of the tinnitus percept, as BK channel activity can regulate input into, and local activity within the AC where sound perception arises (Knaus et al., 1996; Sivaramakrishnan and Oliver, 2001; Thurm et al., 2005; Oliver et al., 2006; Sausbier et al., 2006; Pyott et al., 2007; He et al., 2014; Scott et al., 2017). Several studies support the potential utility of targeting the BK channel to reduce tinnitus (Lobarinas et al., 2011). Lobarinas et al. found that two BK channel openers, Maxipost and its enantiomer, reduced behavioral evidence of salicylate-induced tinnitus in rats using a gap detection task (Lobarinas et al., 2011). Tempering the strength of these findings as support for targeting the BK channel, these compounds also have opposing activity at Kv7 channels; Kv7 is another actively pursued target for preventing tinnitus-related changes in brain activity (Li et al., 2013).

To provide further support for the BK channel as a potential therapeutic target for tinnitus, the studies herein employed a unique BK channel opener, the 1,3,4 oxadiazole, BMS-191011. Originally developed for treating stroke, BMS-191011 has high brain-availability, and higher affinity and specificity for the BK channel than Maxipost (Romine et al., 2007). The current studies showed that systemic treatment with BMS-191011 reduced behavioral manifestation of salicylate-induced tinnitus, but not hyperacusis, in young adult CBA mice. Neurophysiological assessment showed that BMS-191011 treatment did not influence SS-induced changes in auditory brainstem responses (ABRs). But, BMS-191011 did modulate SS-suppressed spontaneous activity in the auditory midbrain following topical administration. Thus, these findings indicate that positive modulation of BK channel function reduces a drug-induced tinnitus percept. Further, the results provide new insights into how BK channel modulation may influence tinnitus—likely by modulating neural activity in the auditory midbrain and cortex. Overall, these findings promote further exploration and development of BK channel openers for reversing central auditory processing deficits associated with tinnitus.

## Materials and methods

### Animals

CBA/CaJ mice, aged 4–8 months, were bred in house and housed 3–4 mice per cage with litter-mates in Sealsafe Plus GM500 cages (36.9 × 15.6 × 13.2 cm) connected to an Aero70 Techniplast Smart Flow system (West Chester, PA, United States). Animal housing was maintained at 24 ± 1°C, with a relative humidity range of 65 ± 3%, and a 12-h light cycle (on at 0700 h). Tap water and mouse chow were available *ad libitum*. All procedures were performed between 0800 and 1,600 h. Study procedures were performed in accordance with the National Institutes of Health’s Guide for the Care and Use of Laboratory Animals, and were approved by the Institutional Animal Care and Use Committee (#IS00000245) at the University of South Florida.

### General procedure for behavioral and auditory brainstem response assays

**FIGURE 1:**
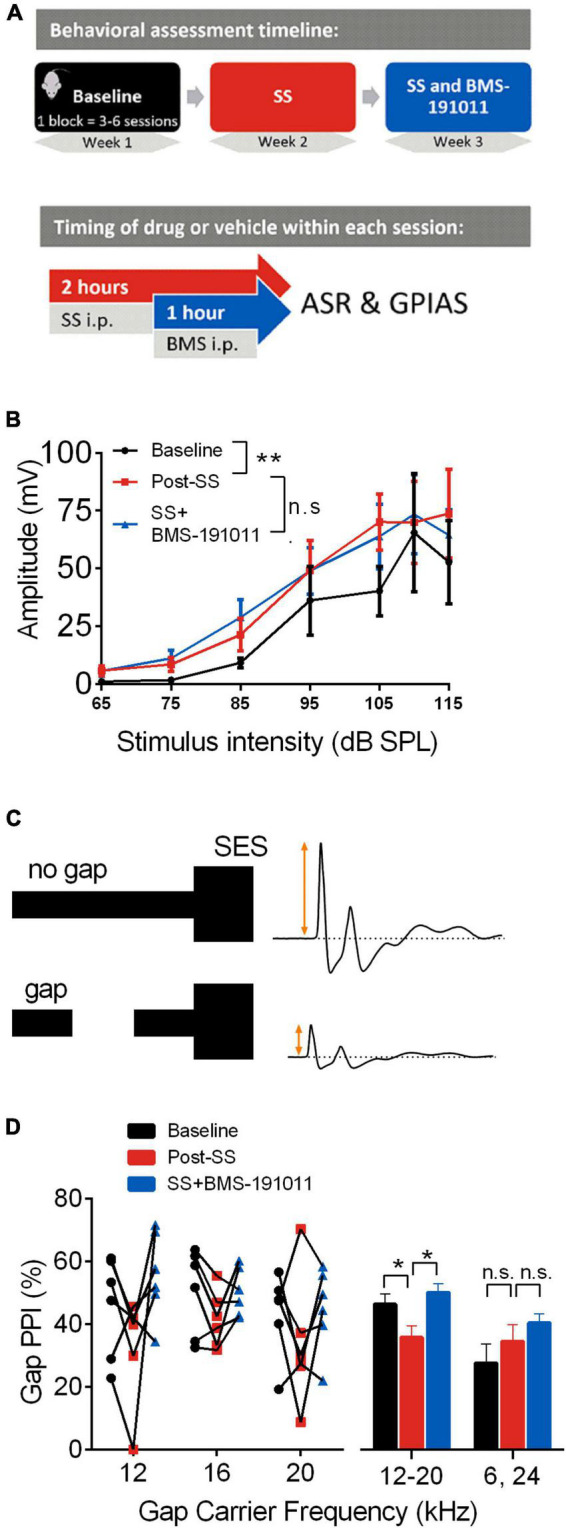
BMS-191011 treatment reduces behavioral manifestations of drug-induced tinnitus. **(A)** Schematic showing the timing of behavioral assessment used to assess the effects of BMS-191011. The behavior of each mouse (*N* = 6) was assessed in sequential blocks of treatment (upper panel). Each treatment block was 3–6 sessions that extended over 1–2 weeks. Within a session (lower panel), SS or vehicle was administered i.p. 2 h prior to assessment, and BMS-191011 or vehicle was administered i.p. 1 h prior to assessment. **(B)** Startle amplitude plotted as a function of SES intensity from 65 to 115 dB SPL. A two-way RM ANOVA indicated a main effect of intensity (*F*(1.217,6.084) = 13.34; *p* < 0.01). Though there was not a main effect of treatment (*F*(1.113,5.563) = 2.8) on startle amplitude, Holm-Sidak *post-hoc* analysis showed a significant increase in startle amplitude relative to baseline (black) following SS administration (orange); this was not significantly altered by co-treatment with BMS-191011 (blue). **(C)** Schematic of the GPIAS assay used to probe the presence of tinnitus. When a 50 ms silent gap is placed prior to the SES, startle amplitude is reduced. Tinnitus reduces the effectiveness of the silent gap in reducing startle amplitude. **(D)** The percent reduction in startle amplitude by a silent gap, or %Gap PPI, displayed for carrier frequencies near the predicted tinnitus frequency region (12–20 kHz) and farther from the predicted tinnitus frequency region (6 and 24 kHz). A two-way RM ANOVA indicated a main effect of treatment (*F*(2,84) = 3.518; *p* < 0.05) and frequency (*F*(1,84) = 9.073; *p* < 0.005). For *post-hoc* analysis, responses to 12–20 kHz carriers were analyzed together, as were responses to 6 and 24 kHz carriers. The left plot shows the %Gap PPI performance for each mouse under each condition. The right plot shows the grouped results of the *post-hoc* analysis. For the 12–20 kHz carriers, there was a significant decrease in %Gap PPI following SS adminsitration (orange) that was returned to baseline levels (black) by co-treatment with BMS-191011 (blue). Responses to 24 kHz carriers are expected to be less influenced by SS-induced tinnitus, and showed no significant change. *N* = 6 mice per group, *p < 0.05, **p < 0.01.

For the primary studies, mice underwent sequential assessment of the effect of treatment in the following dual treatment/control blocks: baseline, sodium salicylate (SS), SS and BMS-191011 (Figure 1A). Corresponding vehicle controls were used in blocks without both SS and BMS-1910111 treatments. In control studies, mice underwent sequential assessment in the following single treatment/control blocks: baseline, BMS-191011. Mice were randomly assigned to study groups probing each primary endpoint, acoustic startle response (ASR) behavior (6 mice), or auditory brainstem responses (ABRs; 12 mice). Three mice were lost to anesthesia before ABRs were measured across all treatments. Two mice were employed in both ASR and ABR studies with a washout period over 2 weeks between. In control experiments, mice underwent sequential assessment in the following single treatment/control blocks: baseline, BMS-191011. ASR behavior was assessed in these control studies (8 mice). Experimental group assignment, data collection, and data analysis were each performed by different investigators. The studies were not preregistered.

Tinnitus was induced transiently by intraperitoneal (i.p.) injection of 250 mg/kg SS. SS induces tinnitus and hyperacusis in humans and animals (Chen et al., 2013; Hickox and Liberman, 2014; Radziwon et al., 2017). Behavioral or neurophysiological manifestations of tinnitus were assessed 2 h following SS injection. To assess how BMS-191011 treatment modulated the impact of SS injection, the compound was administered i.p. (0.5 mg/kg, Sigma Aldrich #SML0866, purchased 2016) 1 h after SS administration, and 1 h prior to testing. In control studies, BMS-191011 was administered alone, 1 h prior to testing.

### Behavioral assays

Behavioral assessment was performed as described previously (Longenecker and Galazyuk, 2011; Lowe and Walton, 2015), and as outlined below. For each treatment block, behavioral responses to auditory stimuli were recorded during at least three testing sessions, every other day, over the course of 1 week. Per block, a total of >30–60 trials were recorded for each stimulus condition, including (1) broadband noises of varying intensity delivered in silence and (2) gap pre-pulse inhibition of the acoustic startle (GPIAS) assessment. The experimenters running the behavioral assessment varied randomly across mice, treatment conditions and studies and were blinded as to which group the mice were assigned to.

The acoustic startle response (ASR), a transient motor response to a startle eliciting stimulus (SES), was converted to voltage by placing mice on a custom-built platform connected to piezoelectric transducers located inside a sound attenuated chamber. Modification of the ASR by stimuli placed before the SES is typically observed as a reduction in startle amplitude, or pre-pulse inhibition (Hoffman and Ison, 1980). To probe the tinnitus percept (Turner, 2007; Longenecker and Galazyuk, 2012), Turner and colleagues have used a relatively long silent gap embedded in an ongoing narrow band noise (NBN) placed before the SES. During this GPIAS paradigm, the silent gap is demonstrably perceived; but, if the tinnitus pitch is in the region of the pitch of the ongoing NBN, the animal will presumably be impaired in detecting the silent gap (Turner et al., 2012). When the center frequency of the NBN is varied during the GPIAS paradigm, the presence of tinnitus is then indicated by a reduction in pre-pulse inhibition to the gap stimulus, or Gap-PPI, when the center frequency of the gap carrier is near the tinnitus pitch (Turner, 2007). All mice administered SS met the inclusion criteria, exhibiting decreased Gap-PPIs for at least one stimulus frequency in response to the GPIAS assay.

### Behavioral testing apparatus

Mice were transported in their home cages to the behavior testing room approximately 30 min before testing. Each animal was handled for at least 5 min before being placed inside a wire mesh cage (9.53 × 3.81 × 4.13 cm), which allowed free movement during testing while maintaining the animal over the center of the cage. Once secured inside the wire mesh cages, animals were placed on top of a platform interfaced to piezoelectric transducers located inside one of eight identical sound attenuated chambers (40.6 × 40.6 × 40.6 cm). Prior to the presentation of the acoustic stimuli, animals were allowed 5 min to acclimate to the testing context.

Acoustic stimuli were presented through Fostex model FT17H speakers (Fostex Company, Tokyo, Japan) located 30 cm directly above the transducer platform and controlled with a RZ6 multi-I/O processor from Tucker-Davis Technologies (TDT, Alachua, FL, United States) and custom MATLAB software (The MathWorks, Inc., Matick, MA, United States). All signals were calibrated prior to testing with a 1/4” microphone placed at the level of the animal’s pinna in the sound chamber and led to a Larsen Davis preamplifier, model 2221 (PCB Piezotronics, Inc., Depew, NY, United States). Transducer responses to movement (in millivolts) were recorded for 125 ms prior and 375 ms following the startle stimulus.

### Behavioral data acquisition

#### Startle i-o

Startle responses were elicited using 20-ms Gaussian broadband noise bursts (1-ms rise/fall time), filtered at 500 Hz–40 kHz. At the start of the behavioral assessment, the intensity of the SES was varied in 10 dB increments at intensities ranging from 55 to 115 dB in order to measure threshold and to acquire the ASR input-output function. For these measures, SESs were presented in a no noise background at random inter-trial intervals between 10 and 20 s.

#### GPIAS

The GPIAS assay was performed approximately 40 min after previous behavioral testing took place. A constant 70 dB Gaussian background NBN (1/3 octave) centered at 6, 12, 16, 20, or 24 kHz was present during GPIAS testing. The SES was delivered at 110 dB SPL during the continuous NBN. During gap trials, a 50-ms silent gap was inserted 100 ms before the SES. Random ISI’s between 10 and 20 s were used.

### Behavioral data processing and statistical analysis

All data processing procedures were built into a custom automated program in R (Grimsley et al., 2015). In this program, peak amplitude of the ASR was defined as the largest positive voltage measurement within 70 ms of the first response peak or 150 ms of SES onset, whichever was shortest. The trial was discarded if the response amplitude in the post-stimulus window was below the mean amplitude plus four standard deviations of the pre-stimulus movement-induced voltage from that same trial. This procedure was shown in previous studies to be the most effective in eliminating non-startle trials (Grimsley et al., 2015). The remaining trials were then averaged for each unique stimulus condition.

Startle data were the arithmetical means derived from all included trials for each animal at the maximum startle amplitude throughout the entire recording (max peak), the latency of the max peak in ms, the latency of the first peak with an amplitude at least 25% of the max peak recording window, root mean-square at baseline from −50 to −1 ms before the SES, and the root mean square of the voltage during the recording time window. Group means were then calculated from these data. Percent pre-pulse inhibition was calculated with the formula [1− (ASR_gap_/ASR_no gap_)] × 100. Startle and Gap PPI functions were analyzed with mixed model ANOVAs with pre-pulse Intensity or Frequency, and Treatment, as factors. Alpha was set at 0.05.

### Auditory brainstem response assays

To obtain an electrophysiological measure of auditory evoked activity in the auditory nerve and brainstem, ABR assays were performed as described previously (Longenecker and Galazyuk, 2011; Lowe and Walton, 2015), and as briefly outlined below. Animals were anesthetized with i.p. administration of ketamine (100 mg/kg) and xylazine (10 mg/kg). This is the Association for Assessment and Accreditation of Laboratory Animal Care’s preferred method of anesthesia for mice. Respiration was monitored during testing and used to guide administration of supplemental anesthesia. Body temperature was kept constant at 37°C using a feedback-controlled heating pad (Physitemp TCAT2-LV Controller, Clifton, NJ, United States). The experimenters running the ABR assays varied randomly across mice, treatment conditions and studies and were blinded as to the which group the mice were assigned to.

Auditory brainstem responses were recorded using TDT System III hardware (RZ6) and BioSig software in a soundproof booth lined with echo-attenuating acoustic foam. ABRs were evoked tone bursts having 3-ms durations (1-ms rise/fall time, alternating polarity) presented at 6, 12, 16, 20, and 24 kHz at a rate of 29/s and averaged for 256 repetitions. Intensity was attenuated in 5 dB steps between 80 and 5 dB SPL. In all cases stimuli were delivered 10–15 dB below the visual detection threshold. Two replicates were obtained at each intensity. Threshold was determined by visual inspection of the lowest stimulus intensity level that produced a defined peak in two replicates. Visual inspection was performed by at least two experimenters who were different than those who collected the data, and agreement within 5 dB was required. Peaks 1-4 (P1-4) were automatically detected and the latency and amplitudes were calculated from each waveform using custom designed MATLAB software.

### Auditory brainstem response data acquisition

Binaural auditory stimuli were generated digitally and delivered *via* a TDT RZ6 Multi-I/O Processor through a multi-field (MF1) magnetic speaker (TDT, Alachua, FL, United States) with a total harmonic distortion = 1% from 1 to 50 kHz. The speaker was centered at 0° azimuth relative to the animal, 10 cm from the pinnae. All stimuli were calibrated using a Larsen Davis preamplifier, model 2221, with a 1/4” microphone and a Larson Davis CAL200 Precision Acoustic Calibrator (PCB Piezotronics, Inc., Depew, NY, United States). ABR signals were acquired using a TDT RA4LI low-impedance digital headstage and RA4PA Medusa preamp with the active (non-inverting) electrode inserted at the vertex, the reference (inverting) electrode below the right ear, and the ground electrode below the left ear. The responses were amplified (20x), filtered (300 Hz—3 kHz), and averaged using BioSig software and the System III hardware (TDT) data-acquisition system.

### Auditory brainstem response data processing and statistical analysis

The peaks were visually verified and corrected, if necessary, to ensure that the identified peak was the last point before the negative slope, as described previously (Allen et al., 2003). Automated analysis measured the peak to trough amplitudes and latencies relative to stimulus onset. Peak amplitudes and latencies to replicate stimuli were first averaged; muscle artifacts exceeding 7 μV were rejected from the averaged response. Peak amplitude ratios were calculated for each animal within testing condition. Group means were then calculated from these data.

### General procedure for IC recordings

Surgery and extracellular recordings were collected and analyzed as described previously (Scott et al., 2017) in three 6-month-old mice. Multi-unit extracellular activity was recorded using vertically oriented single shank silicon acute penetrating 16-channel electrodes with an impedance ranging from 1.2 to 2.1 MΩ (Type-A, 3 mm × 100 μm; NeuroNexus Technologies). To determine the effect of local BMS-191011 administration on drug-induced hyperexcitablity in the IC, multi-channel IC recordings were made in each awake mouse at baseline, 16 h after SS administration, and again 1.5–3.5 h after BMS-191011 was administered. Specifically, baseline multi-channel IC recordings were obtained immediately prior to i.p. administration of 250 mg/kg sodium salicylate (SS). SS induces both behavioral and neural indices of tinnitus (Lowe and Walton, 2015). Free movement was allowed between baseline and post-SS recordings. Recordings were made from several locations to determine the effect of SS (post-SS), and then again at several locations to determine the effect of topical administration of BMS-191011 (1 μL of 10 μM) over the dura. Total spike counts in the excitatory frequency response area (eFRA) was the primary measure that assessed the effect of SS and BMS-191011 administration.

eFRAs from all active channels were acquired simultaneously using 2 to 64 kHz, 25 ms (5 ms rise/fall) tone burst stimuli presented at 0 to 80 dB SPL in 5 dB steps. A total of 2,125 frequency and intensity combinations were presented pseudo-randomly, five times, each at a rate of 10/s. The change in the driven response due to treatment was assessed *via* total spikes within the boundary of each eFRA determined using a custom MatLab GUI. Spontaneous activity was measured by recording 2 min of no-stimulus activity.

### Neurophysiological surgical preparation

To place the multi-channel recording device, the mice were initially anesthetized with an intraperitoneal (i.p.) injection containing 100 mg/kg ketamine and 10 mg/kg xylazine. After anesthesia was induced, the top of the animal’s head and neck was then shaved to prevent contamination of the incision site. The skin was cleaned with germicidal scrub, rinsed with 70% alcohol, and prepped with iodine. The skull was then exposed, 2% lidocaine was applied to the site of incision, and a small brass tube was secured to the skull surface along the sagittal suture at bregma with vet bond and adhered with dental cement. The right IC was located stereotaxically and exposed *via* a small (<1.0 mm) craniotomy. A 24-h recovery period was allowed before beginning the experimental sessions.

### Neurophysiological data acquisition

Prior to making recordings in awake mice, chlorprothixene (Taractin^®^, Roche, 10 mg/kg i.m.) was administered to prevent involuntary movement *via* inhibition of parasympathetic nerve impulses. The animals were then secured in a custom stereotaxic frame (Newport-Klinger) in a heated (34°C) chamber lined with sound-absorbing foam (Sonex^®^). The electrode array was positioned stereotaxically over the right IC in reference to lambda and was advanced dorsoventrally into the IC by a micro positioner (Newport-Klinger PMC 100). The output was attached to a low noise (5–6 μV noise floor) pre-amplifier (RA16), having an operating range of ± 7mV. Neural events were acquired and visualized in real-time using the OpenEx software platform (TDT, Inc.) and a custom designed MATLAB^®^ graphical interface. Neural recordings from each channel were then filtered (300–3,000 Hz), amplified, and sampled at 25 kHz in a 1.25 ms spike-triggered window. A 4:1 signal to noise threshold was automatically set for all channels. A 50-ms broadband noise stimulus presented at 60 dB SPL and a rate of 5/s was used to estimate spike-trigger thresholds. Each penetration typically yielded 8–13 active channels. Recording sessions lasted less than 6 h, and if at any time a mouse showed signs of discomfort, such as excessive movement, it was removed from the apparatus and testing was halted.

Noise and tone bursts were generated digitally (Real-time Processor Visual Design Studio, TDT) using a System 3 processor and D/A converter (TDT RX6) with 200 kHz sampling rate. The signals were routed to an electrostatic speaker (TDT ES1) with a flat frequency response from 4 to 110 kHz. This speaker was placed at 60° azimuth contralateral to the recording site. Harmonic distortions were measured with a Dynamic Signal Analyzer (HP 35665A) and were at least 60 dB below the primary signal. The distance between the speaker and the pinna was fixed at 22.5 cm and calibrated using a B&K 2610 amplifier and a 1/4” microphone placed at the location of the pinna.

### Neurophysiological data processing and statistical analysis

Spike waveforms were processed in MATLAB^®^ using the TDT OpenDeveloper ActiveX controls and passed to AutoClass C v3.3.4, an unsupervised Bayesian classification system that seeks a maximum posterior probability classification, developed at the NASA Ames Research Center (Stutz and Cheeseman, 1996; Cheeseman and Stutz, 1997). AutoClass scans the dataset of voltage–time waveforms according to custom specified spike parameters to produce the best-fit classifications of the data, which may include distinct single- and multi-unit events, as well as noise. To discriminate the signal from noise, the variance of the background noise was estimated as the quartile range of the first five digitization points of the spike waveform, as these are recorded prior to the threshold-crossing event. To avoid overloading AutoClass with excessive noise, which leads to over-classification, this noise measure was used to screen the event waveform data such that only voltage points with absolute values greater than this noise floor were presented for use in the classification. Once the classes had been determined in each channel of data, they were visualized within a custom MATLAB^®^ program and assigned to multi-unit, single-unit, or noise classes. Event classes that were categorized as noise were subsequently discarded, and units with distinct biphasic waveforms and good SNR were classified as single-units. This was performed by an experimenter blind to condition. As most channels recorded information elicited from the spiking of two or more neurons, all recordings in this paper were considered to be multi-unit activity (Kirby and Middlebrooks, 2012). Nonetheless, there was no observation of any consistent differences in the eFRAs between single units and multi-unit clusters.

eFRAs were analyzed using a custom MATLAB^®^ program by an experimenter blind to condition. The frequency at which driven activity is responsive at the lowest intensity (threshold) is classified as the characteristic frequency (CF) and the point in the receptive field which elicits the maximal driven activity is categorized as the best frequency (BF). A custom MATLAB^®^ program was used to calculate the edges of each channel’s eFRA, and this was verified *via* visual inspection to ensure no non-driven activity was included in the calculation. The edges of the eFRA were defined as the activity levels that were equal to or greater than the background rate and at least 15% of the maximum rate. The change in the driven response due to treatment was assessed *via* spike rate-level functions derived from within the boundary of each eFRA, that is, responses to CF tones presented from 0 to 80 dB SPL, in 5 dB steps. The maximum number of spikes per stimulus were calculated for this stimulus set.

### Statistics

Data are presented as mean ± SEM. Statistical analysis was performed in GraphPad (GraphPad Software, La Jolla, CA, United States). Two-way or one-way ANOVA and Holm-Sidak *post-hoc* analysis evaluated the effects of treatment. A Shapiro–Wilk test was employed to confirm normality. *N*s are presented in the figure legends. Alpha was set at 0.05 for all statistical tests.

## Results

Acoustic startle response assays were utilized to probe central auditory processing changes before and after SS administration in young adult CBA/CaJ mice. Modification of the acoustic startle reflex (ASR), elicited by wideband noise stimuli, provided a functional readout of changes in sensorimotor gating. First, startle amplitude was measured in response to multiple intensities of an acoustic startle elicitor. As expected (Lowe and Walton, 2015), SS administration increased the intensity-response function relative to baseline (Figures 1A,B). To test whether the systemic administration of the BK channel opener, BMS-191011, suppressed the SS-induced increase in startle amplitudes, mice were administered both SS and BMS-191011 before behavioral testing. BMS-191011 treatment did not reverse the effects of SS administration on the startle amplitude intensity-response function (Figure 1B). BMS-191011 treatment also did not alter startle amplitudes when administered alone (Supplementary Figure 1B). Thus, BMS-191011 does not alter baseline sensorimotor gating, nor SS-augmented sensorimotor responses suggesting enhanced acoustic sensitivity. This latter indication of hyperactivity in central auditory processing is consistent with the expected presence of hyperacusis following SS administration (Eggermont, 2013; Lobarinas et al., 2013; Radziwon et al., 2017).

Next, modification of ASRs by acoustic pre-pulse stimuli probed the presence of tinnitus following SS administration. When an audible stimulus occurs shortly (e.g., 100 ms) before the startle elicitor, the ASR is inhibited, and this is referred to as pre-pulse inhibition. In the GPIAS paradigm (Figure 1C), a silent gap in noise inhibits the startle response relative to a no-gap condition (Gap-PPI). The degree to which the ASR amplitude is reduced is directly related to the salience of the gap pre-pulse. Failure of the silent gap to suppress a subsequently elicited startle reflex, indicated by a reduction in Gap-PPI, is thus indicative of a tinnitus percept near the pre-pulse frequency (Turner, 2007; Longenecker and Galazyuk, 2012). As expected, SS administration reduced Gap-PPI relative to baseline when the silent gap was placed in 12, 16, or 20 kHz carriers (Figure 1D). This indicates that SS administration induced tinnitus percepts in the 12–20 kHz range. Administration of BMS-191011 returned SS-suppressed Gap-PPIs to baseline levels for these carrier frequencies (see also Supplementary Figure 1A). In contrast, SS administration did not significantly influence Gap-PPI for 6 and 24 kHz carriers (Figure 1D and Supplementary Figure 1A), outside of the expected frequency range of the tinnitus percept (Bauer et al., 1999; Chen et al., 2012; Stolzberg et al., 2012; Lowe and Walton, 2015). Additionally, Gap-PPI functions were not significantly altered from baseline when BMS-191011 treatment was administered alone (Supplementary Figure 1C). Together, these findings indicate that BMS-191011 treatment reduces behavioral manifestation of SS-induced tinnitus.

**FIGURE 2:**
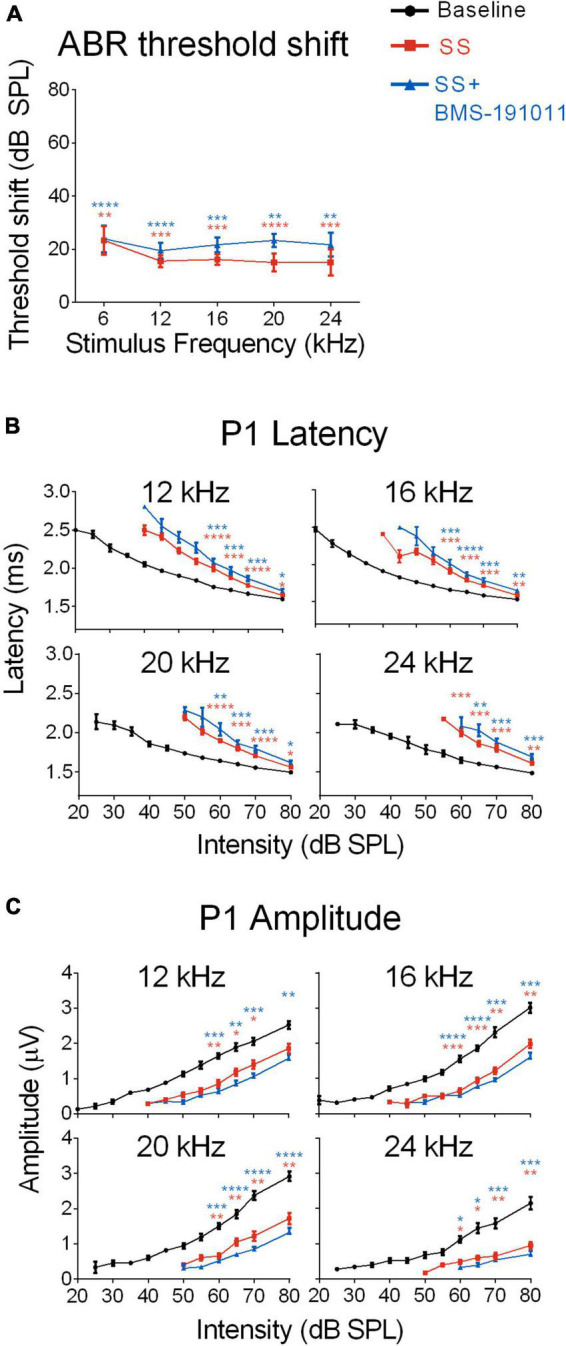
BMS-190111 treatment does not effect SS-suppressed auditory nerve output. **(A)** ABR stimulus frequency ploted as a function of threshold shift relative to baseline for post-SS and post- SS + BMS. A two-way RM ANOVA of threshold shifts showed a main effect of treatment (*F*(1.233,9.866) = 38.98; *p* < 0.0001) and frequency (*F*(1.681,13.45) = 52.26; *p* < 0.0001) when SS (orange) and SS + BMS-191011 (blue) groups were included. *Post-hoc* analyses showed that thresholds increased relative to baseline following SS administration, but were not further altered by BMS-191011 treatment. Orange and blue asterisks indicate a significant difference from baseline for the SS and SS + BMS-191011 groups, respectively. **(B)** Plots show the quantification of P1 latencies as a function of intensity for 12, 16, 20 and 24 kHz tone bursts at baseline (black), post-SS (orange) and post-SS + BMS (blue). Two-way RM ANOVAs indicated a main effect of treatment for all frequencies (12 kHz: *F*(1.629,17.92) = 31.53; *p* < 0.0001; 16 kHz: *F*(1.926,21.19) = 40.95; *p* < 0.0001; 20 kHz: *F*(1.717,18.89) = 33.40; *p* < 0.0001; 24 kHz: *F*(1.663,16.63) = 36.64; *p* < 0.0001). There was also a main effect of intensity (12 kHz: *F*(1.079,11.87) = 368.7; *p* < 0.0001; 16 kHz: *F*(1.051,11.56) = 291.2; *p* < 0.0001; 20 kHz: *F*(1.132,12.46) = 320.6; *p* < 0.0001; 24 kHz: *F*(1.434,14.34) = 217.2; *p* < 0.0001). *Post-hoc* analyses indicated that SS treatment significantly delayed P1 latencies relative to baseline (black) when administered alone (orange asterisks) or with BMS-191011 (blue asterisks). In contrast, BMS-191011 treatment minimally altered the effect of SS administration. **(C)** P1 amplitudes plotted as as a function of intensity for 12, 16, 20, and 24 kHz tones. Two-way RM ANOVAs indicated a main effect of treatment for all frequencies (12 kHz: *F*(1.637,11.46) = 15.29, *p* < 0.001; 16 kHz: *F*(1.703,11.92) = 46.48, *p* < 0.0001; 20 kHz: *F*(1.351,9.460) = 37.97, *p* < 0.0001; 24 kHz: *F*(1.181,8.266) = 30.50, *p* < 0.001). There was also a main effect of intensity (12 kHz: *F*(1.570,10.99) = 126.6; *p* < 0.0001; 16 kHz: *F*(1.718,12.03) = 111.5; *p* < 0.0001; 20 kHz: *F*(1.635,11.44) = 99.33; *p* < 0.0001; 24 kHz: *F*(1.237,8.662) = 41.57; *p* < 0.0001). *Post-hoc* analyses indicated that SS treatment significantly reduced P1 amplitudes relative to baseline when administered alone (orange asterisks) or with BMS-191011 (blue asterisks). BMS-191011 treatment did not significantly alter the effect of SS administration. *N* = 8–12 mice per group, *p < 0.05, **p < 0.01, ***p < 0.001, ****p < 0.0001.

Although SS administration reduces cochlear sensitivity, sound-evoked signals are increased in the cochlear nucleus, influencing sound processing in ascending CAS structures (Jastreboff and Sasaki, 1986; Chen and Jastreboff, 1995; Kaltenbach et al., 2005; Chen et al., 2013; Lowe and Walton, 2015). To determine whether BMS-191011 treatment alters these neurophysiological processes, ABRs were recorded before and after SS administration to observe changes in cochlear and brainstem auditory processing. SS administration produced the expected broad-spectrum reduction in cochlear responses (Chen and Jastreboff, 1995), indicated by increased ABR thresholds, by ∼20 dB, across the frequency spectrum (Figure 2A and Supplementary Figure 2A). Reduced cochlear sensitivity following SS administration was further indicated by longer P1 latencies, particularly at lower intensities, and reduced P1 amplitudes relative to baseline (Figures 2B,C). As expected, latency and threshold covary such that threshold-normalized latencies overlapped for all treatment groups (Supplementary Figure 2B). When BMS-191011 was administered after SS, 1 h before ABR recordings, BMS-191011 treatment did not significantly alter SS-induced changes in ABR thresholds, P1 latencies or P1 amplitudes (Figures 2B,C). Thus, BMS-191011 treatment did not significantly impact the SS-induced decrease in cochlear thresholds.

**FIGURE 3:**
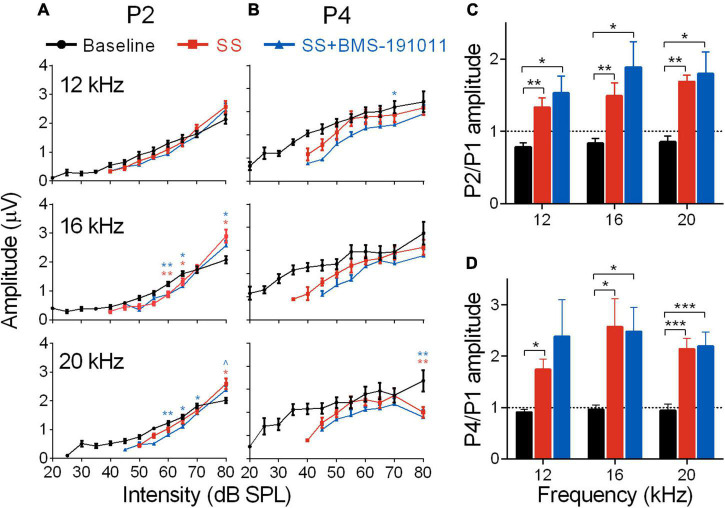
BMS-191011 treatment does not suppress the increased gain in early auditory structures caused by SS treatment. **(A)** Plots show the quantification of P2 amplitudes as a function of intensity for 12, 16, and 20 kHz tones. Two-way RM ANOVAs indicated a main effect of intensity for all frequencies (12 kHz: *F*(1.278,14.06) = 167.6, *p* < 0.0001; 16 kHz: *F*(1.117,12.28) = 203.2, *p* < 0.0001; 20 kHz: *F*(1.823,20.06) = 187.1, *p* < 0.0001), but not for treatment (12 kHz: *F*(1.771,19.48) = 0.2766; 16 kHz: *F*(1.358,14.94) = 0.4457; 20 kHz: *F*(1.322,14.54) = 0.9917). There was a significant interaction of treatment and intensity for all frequencies (12 kHz: *F*(1.847,14.16) = 5.916, *p* < 0.05; 16 kHz: *F*(2.145,16.45) = 14.85, *p* < 0.001; 20 kHz: *F*(3.022,23.17) = 9.327, *p* < 0.001). *Post-hoc* analyses indicated that, relative to baseline, SS administration increased growth in the intensity-response function, with larger amplitudes at 80 dB, particularly at 16 kHz. Orange asterisks compare SS administration alone with baseline, and blue symbols compare SS + BMS-191011 treatment with baseline. BMS-191011 treatment did not significantly alter the effect of SS administration. **(B)** Plots show the quantification of P4 amplitudes as a function of intensity for 12, 16 and 20 kHz tones. Two-way RM ANOVAs showed no main effect of treatment (12 kHz: *F*(1.735,19.08) = 1.532; 16 kHz: *F*(1.665,18.32) = 1.246; 20 kHz: *F*(1.714,18.85) = 2.712), but either showed a significant main effect of intensity (12 kHz: *F*(2.023,22.25) = 3.573, *p* < 0.05; 16 kHz: *F*(1.199,13.19) = 6.916, *p* < 0.05; 20 kHz: *F*(1.467,16.13) = 0.7032), or a significant interaction between treatment and intensity (12 kHz: *F*(3.338,25.59) = 0.0996; 16 kHz: *F*(2.988,22.90) = 0.8136; 20 kHz: (*F*(3.139,24.07) = 3.433; *p* < 0.05). *Post-hoc* analysis indicated similar responses across treatment groups for 12 and 16 kHz stimuli. For 20 kHz stimuli, significant suppression occurred at the upper end of intensity. **(C)** Mean amplitude of P2 relative to P1 for 60 dB tones. A two-way RM ANOVA showed a main effect of treatment on gain in the auditory brainstem (*F*(1.171,12.88) = 20.80; *p* < 0.0001), but not frequency (*F*(1.80,19.81) = 1.776). *Post-hoc* analysis indicated that both SS administration alone (orange) and with BMS-191011 treatment (blue) significantly increased the P2/P1 ratio relative to baseline (black). **(D)** A graph of the amplitude of P4 relative to P1 for 60 dB tones. Consistent with the P2/P1 ratios, a two-way RM ANOVA also show an effect of treatment (*F*(1.1801,19.81) = 16.81; *p* < 0.0001), but not intensity (*F*(1.19,13.09) = 0.9818) on gain in the auditory brainstem. *Post-hoc* analyses indicated that both SS administration alone (orange) and with BMS-191011 treatment (blue) significantly increased the P4/P1 ratio relative to baseline (black) at all intensities. Dotted lines at P2/P1 and P4/P1 = 1 demarcate equal amplitudes. *N* = 8–12 mice per group, *p < 0.05, **p < 0.01, ***p < 0.001.

Despite reduced cochlear sound sensitivity, tinnitus is associated with amplified sound-evoked activity near the tinnitus percept in brainstem auditory structures for both humans (Gu et al., 2012) and SS-treated mice (Lowe and Walton, 2015). To measure activity in the brainstem through lemniscal pathways, ABR P2 and P4 amplitudes were measured and compared with AN output (P1). In response to SS administration, P2 amplitudes for 12, 16, and 20 kHz stimuli had suppressed, but steeper intensity-response functions. At a high intensity (80 dB) P2 amplitudes were larger than baseline, particularly at 16 kHz (Figure 3A). P4 amplitudes were not significantly different from baseline for 12–16 kHz stimuli (Figure 3B). In contrast, for 6 and 24 kHz stimuli both P2 and P4 amplitudes showed suppression that remained constant or increased with intensity (Supplementary Figures 3A,B). The ratios of P2 to P1 amplitudes were substantially increased for 12–20, but not 6 and 24 kHz stimuli (Figure 3C and Supplementary Figure 3C). The increase was maintained for P4/P1 amplitude ratios for the central frequencies (Figure 3D and Supplementary Figure 3D). Treatment with BMS-191011 did not substantively alter these effects of SS administration (Figures 3C,D).

**FIGURE 4:**
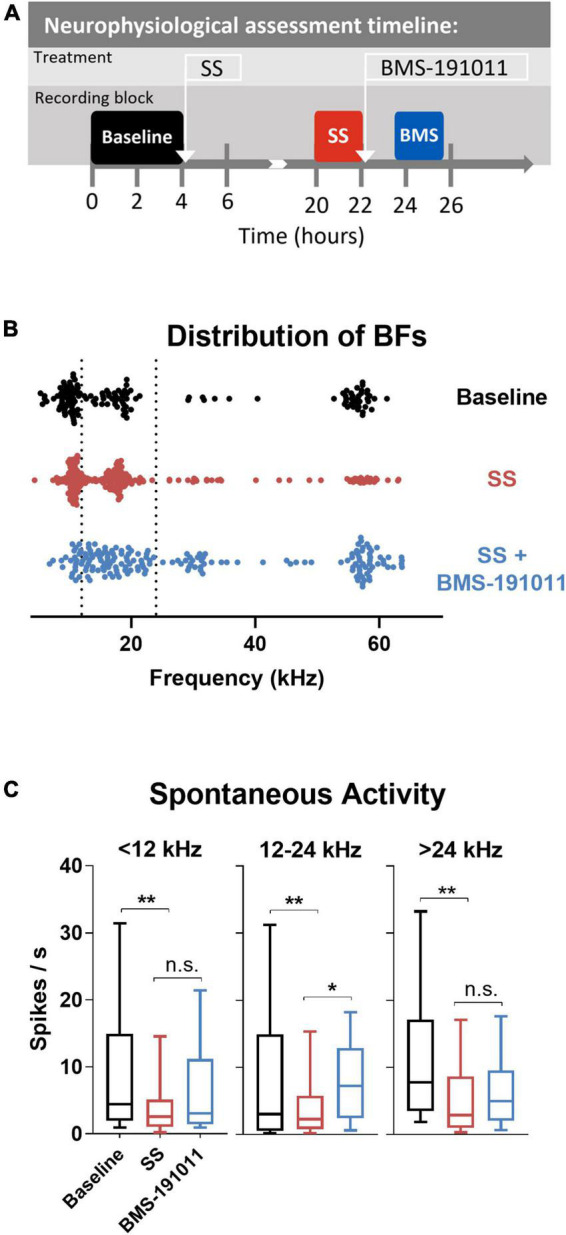
BMS-191011 treatment reverses spontaneous activity induced by sodium salicylate in the inferior colliculus. **(A)** Schematic showing the timing of the neurophysiological recordings included in the analysis. Recordings were made prior to I.P. administration of SS (baseline-black), after systemic SS administration (orange), and again after administration of BMS-191011 applied locally over the IC (blue). Between baseline recordings and SS recordings, free movement was allowed in the home cage. Multiple penetrations of the multi-electrode array were used for each timepoint in order to maximize the number of active channels. In the last location neural activity was measured before and after BMS-191011 administration allowing the SS and SS + BMS-191011 effects to be observed in the same neurons. **(B)** Plot of the best frequencies (BFs) for each recorded IC unit at baseline, after SS administration, and again after BMS-191011 application. The dotted lines demarcates the grouping of units in the subsequent panels. **(C)** Plots of mean spontaneous spike rates measured at baseline (black), the day after SS administration (orange), and again after BMS-191011 administration (blue). One-way ANOVAs showed a significant effect of treatment for units with BFs < 12 kHz (*F*(2,170) = 4.419; *p* < 0.05), 12 – 24 kHz (*F*(2,242) = 6.798; *p* < 0.01), and >24 kHz (*F*(2,170) = 5.403; *p* < 0.001). *Post-hoc* analyses indicated that SS administration decreased spontaneous activity in each frequency group. Subsequent local BMS-191011 application then limited this impact of SS in the 12 – 24 kHz units. *p < 0.05, **p < 0.01.

**FIGURE 5:**
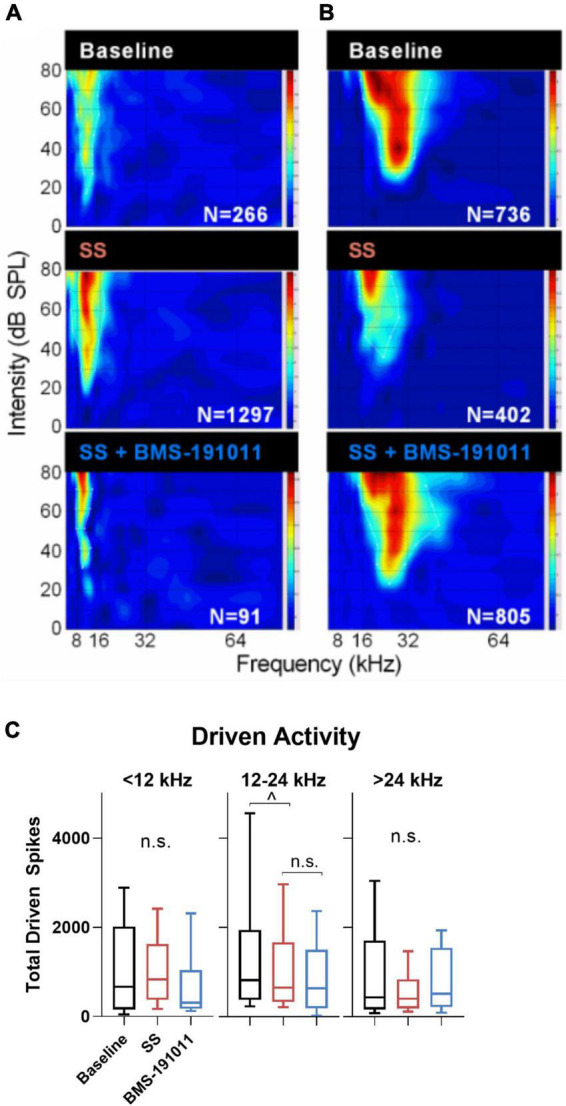
Changes in evoked activity occurred in some, but not all IC units following SS and BMS-191011 administration. **(A,B)** Representative recordings are shown for two units in the IC. eFRAs are shown for baseline, the day after SS administration, and again after BMS-191011 administration. Spike counts (Ns) were measured within the excitatory boundaries of the eFRA and reported at each timepoint. Some lower frequency units showed an increase in activity following SS administration that was subsequently suppressed by BMS-191011 application. Some mid-to-high frequency units showed decreases in activity following SS administration that was recovered following BMS-191011 application. Neither pattern was consistent enough to drive significantly changes in mean driven activity. **(C)** Plots of mean total driven spike counts measured from the eFRAs at baseline (black), the day after SS administration (orange), and again after BMS-191011 administration (blue). One-way ANOVAs did not show clear effects of treatment. The one-way ANOVA was not significant for units with BFs ≤ 12 kHz or >24 kHz (*F*(2,194) = 1.362; *p* = 0.26; *F*(2,175) = 2.397; *p* = 0.09). The one-way was significant for units with mid-range BFs (*F*(2,181) = 4.140; *p* = 0.0175), but this appeared to be driven predominantly by effects of SS, but not BMS-191011. Additionally, subsequent Holms-Sidak *post-hoc* analyses did not show significant differences between time points for these mid-range units.

Though BMS-191011 treatment did not suppress the enhancement in central gain in brainstem auditory structures measured with the ABR after SS administration, these two drugs may comodulate activity in higher CAS regions. Previous work has shown that either systemic SS administration (Jastreboff and Sasaki, 1986; Chen and Jastreboff, 1995; Kaltenbach et al., 2005; Chen et al., 2013; Lowe and Walton, 2015) or local application of BK channel modulators (Sausbier et al., 2006; Scott et al., 2017) can influence neuronal excitability in the IC. To test the possibility that local BK channel function can influence SS-induced changes in the IC, multi-channel neurophysiological recordings were made in the IC before and after systemic SS administration, and again to explore the impact of local BMS-191011 treatment (Figure 4A). The best frequency (BF) of each unit was determined from the eFRA (Figure 4B), and units were grouped by BF, <12, 12–24, and >24 kHz. SS administration reduced representation of the highest BFs between 50 and 65 kHz. After application of BMS-191011 over the midbrain, representation of high frequency BFs appeared similar to baseline (Figure 4B). Enhanced high frequency representation following local BMS-191011 administration over the IC was also apparent in animals that had not received SS (Figure 5A). To quantify changes in spontaneous activity that may support the rise of the tinnitus percept, the number of spikes was assessed in quiet during a 2-min period. There was an effect of treatment on the spontaneous activity of IC neurons across the tonotopic axis (*F*(2,602) = 14.02; *p* < 0.0001). *Post-hoc* analysis indicated that SS administration suppressed spontaneous activity (*p* < 0.0001), while dural BMS-191011 administration increased spontaneous activity (*p* < 0.05). When spontaneous activity was observed in IC neurons grouped by BF, similar findings emerged, particularly in the mid-frequencies (Figure 4C). SS administration suppressed spontaneous activity in each frequency channel. For IC neurons with BFs between 12 and 24 kHz, spontaneous activity was then increased by BMS-191011 application. Thus, in the frequency range of the tinnitus percept, errant decreases in spontaneous network activity driven by SS are ameliorated by local BMS-191011 administration. In contrast with the effect of BMS-191011 in the SS-exposed IC, BMS-191011 applied alone at the same concentration caused no change in spontaneous activity for neurons with BFs in the low- to mid-frequency range (Figure 4B), and decreased spontaneous activity for neurons with high-frequency BFs (Figure 4B). For additional comparison, evoked activity was assessed by measuring the total number of spikes within excitatory frequency-response areas (eFRA) for units grouped by BF, <12, 12–24, and >24 kHz. Some lower frequency (<12 kHz, Figures 5A,C) units showed an increase in activity following SS administration that was subsequently suppressed by BMS-191011 application while some mid-to-high frequency (≥12 kHz, Figures 5B,C) units showed decreases in activity following SS administration that was recovered following BMS-191011 application. However, these changes in evoked activity were less consistent than for spontaneous activity. Together these findings indicate that local administration of BMS-191011 in the IC rebalances aspects of SS-induced neural plasticity, particularly as related to spontaneous activity.

Overall, these data indicate that BMS-191011 treatment counteracts a behavioral manifestation of SS-induced tinnitus, but not a behavioral measure of hyperacusis. SS administration induced the expected increased gain in sound-evoked responses for brainstem auditory regions associated with tinnitus and hyperacusis. But, BMS-191011 treatment did not alter this neurophysiological marker. Instead, local administration of BMS-191011 ameliorated SS-induced suppression of spontaneous activity in the auditory midbrain in the frequency range of the tinnitus percept. These findings support the ability of BMS-191011 treatment to counteract tinnitus-associated changes in CAS neurophysiology, which may include local action in the auditory midbrain.

## Discussion

The studies reported herein explored the effects of systemic administration of the BK channel opener, BMS-191011, on behavioral and neurophysiological biomarkers of tinnitus and hyperacusis. Salicylate administration reliably produces a narrowband tinnitus percept and hyperacusis in rodents, which coincide with enhanced gain in the CAS despite raised cochlear thresholds (Yang et al., 2007; Stolzberg et al., 2012; Lowe and Walton, 2015). In the present study, an acoustic startle response behavioral read-out of central auditory function showed that salicylate administration induced the expected tinnitus percept, roughly centered around 16 kHz (Bauer et al., 1999; Chen et al., 2012; Stolzberg et al., 2012; Lowe and Walton, 2015), which was reduced by treatment with BMS-191011. Interestingly, BMS-191011 did not influence a behavioral measure of CAS hyperactivity more indicative of hyperacusis than tinnitus, enhanced acoustic startle responses. Neurophysiological assessment showed that BMS-191011 treatment did not substantially influence the salicylate-induced increase in cochlear thresholds nor enhancement in central gain in the auditory brainstem. Instead, local administration of BMS-191011 over the IC normalized altered spontaneous responses in mid-frequency midbrain neurons following salicylate administration. While spontaneous activity in the CAS is not necessarily a direct measure of tinnitus, alterated activity for neurons with BFs between 12 and 24 kHz, in the range of the tinnitus percept, may capture changes in network activity that are necessary for the tinnitus percept to arise in the auditory cortex (AC). Thus, these findings suggest that BMS-191011 treatment may suppress salicylate-induced tinnitus by shaping network activity in the CAS, with evident influences in the auditory midbrain.

Evidence that BK channel function might influence drug-induced tinnitus was first indicated by preclinical studies with exemplary 3-fluorooxindoles. A set of these compounds were initially developed by Bristol-Myers Squibb as BK channel openers, but later found to be more potent KCNQ channel modulators (Korsgaard et al., 2005). Lobarinas et al. (2011) examined the effect of two compounds in this class, Maxipost and its enantiomer, and found that both suppressed behavioral evidence of salicylate-induced tinnitus in rats. Despite opposing action at the KCNQ channel, Maxipost and its enantiomer are both positive modulators (i.e., openers) of the BK channel. Continuing to support the utility of BK channel openers in reducing tinnitus, the current findings show that an oxadiazolone-class compound, with higher BK channel affinity and specificity than 3-fluorooxindoles (Romine et al., 2007), also reduced behavioral manifestations of salicylate-induced tinnitus. One potential confound in interpreting these findings is that both studies have employed an acoustic startle gap detection assay to probe the presence of SS-induced tinnitus. Our findings and others show enhanced ASRs after salicylate administration, which have been linked to hyperacusis (Radziwon et al., 2017). Heightened responses could reduce suppression of the ASR in the gap detection assay, i.e., the behavioral readout of tinnitus (Brozoski and Bauer, 2016). However, BMS-191011 administration reduces the GPIAS measure of tinnitus without suppressing ASR functions, indicating that in this model measures of tinnitus and hyperacusis are differentiable, and that BMS-191011 more strongly influences the former.

Salicylate-induced tinnitus and hyperacusis are associated with altered neural activity starting in the cochlear nucleus, and ascending into the auditory midbrain (Jastreboff and Sasaki, 1986; Chen and Jastreboff, 1995; Kaltenbach et al., 2005; Chen et al., 2013; Lowe and Walton, 2015). Previous studies have found that salicylate modulates the spontaneous activity of midbrain neurons. *In vitro* application of salicylate decreases spontaneous firing in IC neurons recorded in mouse midbrain slices (Basta and Ernst, 2004). Studies exploring the effects of systemic salicylate administration *in vivo* have shown varied changes in spontaneous activity across the tonotopic regions of the IC (Jastreboff and Sasaki, 1986; Chen and Jastreboff, 1995; Ma et al., 2006). A study in CBA mice found that spontaneous firing increased in low and mid frequency neurons, and decreased in high frequency neurons following systemic salicylate administration (Ma et al., 2006). This latter finding differs from the present study, where spontaneous activity decreased across the tonotopic axis. Interestingly, our results indicate that local BMS-191011 application can normalize these changes in IC activity after systemic salicylate administration, particularly in the frequency region of the tinnitus percept. While neurophysiological changes in ascending lemniscal and non-lemniscal auditory pathways, and projections from non-auditory areas like the limbic system (Kaltenbach et al., 2005; Rauschecker et al., 2010; Norena, 2011; Henry et al., 2014), all feed into the AC to generate the tinnitus percept, this local influence of BMS-191011 in the IC may be one way that systemic treatment contributes to reducing behavioral manifestations of tinnitus.

Positive modulation of BK channel function can enhance neural excitability (N’Gouemo, 2011), as is found herein following suppression of spontaneous activity by SS administration. BK channels appear well situated to impact neural activity subserving the tinnitus percept as the expression patterns allow regulation of input into and local activity within the AC, where sound perception arises. In the cochlea, the BK channel contributes to the repolarizing current in hair cells (Thurm et al., 2005; Oliver et al., 2006; Pyott et al., 2007). In the CAS, the BK channel is expressed in brainstem, modulating signaling in the dorsal cochlear nucleus and at the calyx of Held (He et al., 2014). The IC has moderate to high BK channel expression where it shapes action potential firing (Sausbier et al., 2006) and sound-evoked activity (Scott et al., 2017). Finally, the AC has high expression levels of both BK channel transcripts and protein (Knaus et al., 1996). In addition to this neuronal expression, cerebrovascular BK channels could subtly influence cochlear or neural activity subserving the tinnitus percept. In this study, BMS-191011 treatment did not alter cochlear sensitivity or brainstem responses to tones, or strongly alter spontaneous activity in IC neurons when administered alone; but, BMS-191011 treatment did normalize SS-induced changes in spontaneous activity in the IC. These findings are consistent with our previous finding that BK channel modulation did not strongly influence sound-evoked activity in the periphery and brainstem (Scott et al., 2017). Other studies also suggest that BK channel activation ameliorates dysregulation of neural activity in the IC, including dysregulation consequent to noise exposure, and for tinnitus-like hyperactivity in an *in vitro* neuronal network derived from mouse AC (Wu et al., 2014).

To better test the translational potential of BMS-191011 treatment for tinnitus, it will be important to employ other models of tinnitus induction. The etiology is mixed for patients with tinnitus, but noise exposure is the most common underlying contributing factor (Henry et al., 2014). There are clear differences in the mode of peripheral damage and the outcome of acute vs. chronic tinnitus between salicylate toxicity and acoustic trauma. Nonetheless, some changes in CAS function subserving the tinnitus percept are likely similar between these modes of induction, including enhanced central gain (Salvi et al., 2000; Lanting et al., 2009; Norena, 2011). Thus, the ability of BMS-191011 treatment to reduce the behavioral manifestation of tinnitus and normalize auditory midbrain activity after salicylate administration support further exploration of new drug candidates that target the BK channel for treating tinnitus. Compounds with similar activity to BMS-191011 hold promise for optimized drug candidates. Though BMS-191011 itself has low aqueous solubility and oral bioavailability, structure-activity relationship studies have explored analogs and cleavable prodrugs with the goal of improving solubility while maintaining high brain availability and specific BK channel activity (Romine et al., 2007). Since other pharmaceuticals with BK channel opener activity are used safely in humans (N’Gouemo, 2011), the class of compounds exemplified by BMS-191011 holds potential for treating individuals with tinnitus.

## Data availability statement

The raw data supporting the conclusions of this article will be made available by the authors, without undue reservation.

## Ethics statement

The animal study was reviewed and approved by the University of South Florida IACUC.

## Author contributions

LS and JW: conception and design, interpretation of data, and writing the manuscript. AL, EB, LF-W, LS, and JW: collection and analysis of data. All authors contributed to the article and approved the submitted version.
