# Supplementary material for: Small molecule modulation of the large-conductance calcium-activated potassium channel suppresses salicylate-induced tinnitus in mice
Source: Front Neurosci. 2022 Aug 25;16:763855. doi: 10.3389/fnins.2022.763855 (PMC9453485; doi:10.3389/fnins.2022.763855)
Supplement: Supplementary file 1 [file Data_Sheet_1.pdf]

Supplemental Figures

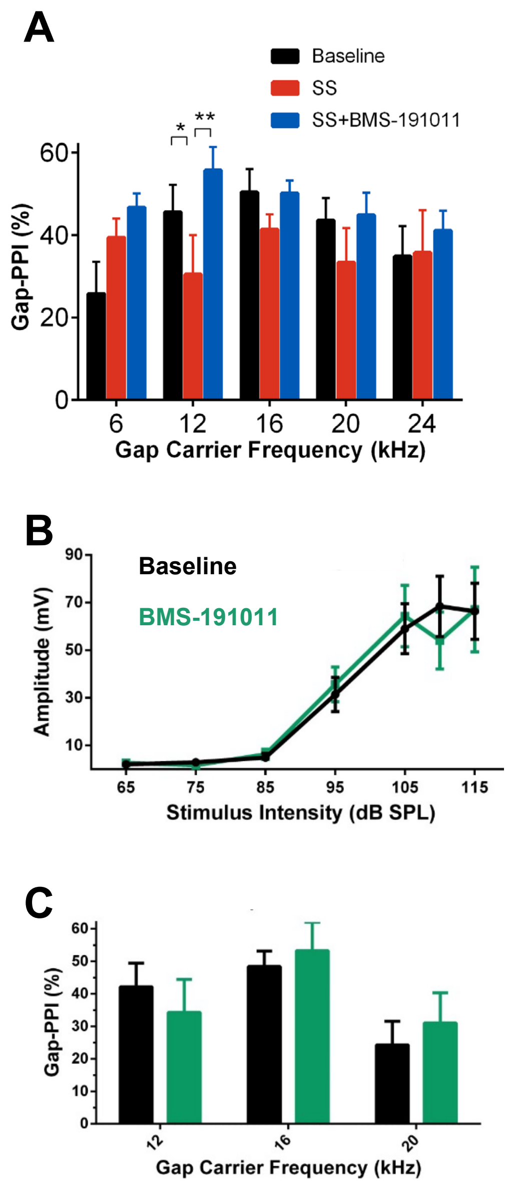

Supplemental Figure 1. **BMS-191011 treatment alters the effects of SS administration but does not alter baseline ASR functions. (A)** The percent reduction in startle amplitude by a silent gap, or %Gap PPI, displayed for each carrier frequency for the data in Figure 1D. The %Gap PPI was measured at baseline (black), after SS administration (orange), and after SS+BMS-191011 (blue; see Figure 1A for study details). When each carrier frequency was entered individually, a two-way RM ANOVA indicated a main effect of treatment ($F(2, 10) = 13.80$; $p<0.005$), but not frequency ($F(4, 20) = 0.78$). Post-hoc analysis showed a significant decrease in %Gap PPI following SS adminsitration (orange) that was returned to baseline levels (black) by co-treatment with BMS-

191011 (blue) for the 12 kHz carrier. *N*=6 per group, *$p \leq 0.05$, **$p < 0.01$. **(B)** ASR functions were measured in a separate study assessing the effects of BMS-191011 treatment alone. Assessments were made during a baseline block (black) and again during a BMS-191011 treatment block (green). Startle amplitude is plotted as a function of SES intensity. A two-way RM ANOVA showed a main effect of intensity ($F(6, 84) = 32.72$; $p < 0.0001$). There was no significant effect of BMS-191011 treatment alone (green) on baseline (black) startle amplitudes ($F(1, 14) = 0.003$). **(B)** The percent reduction in startle amplitude by a silent gap, or percent Gap PPI, displayed for several carrier frequencies. A two-way RM ANOVA showed that there was not a significant effect of treatment ($F(1, 7) = 0.06$). *N*=8 per group.

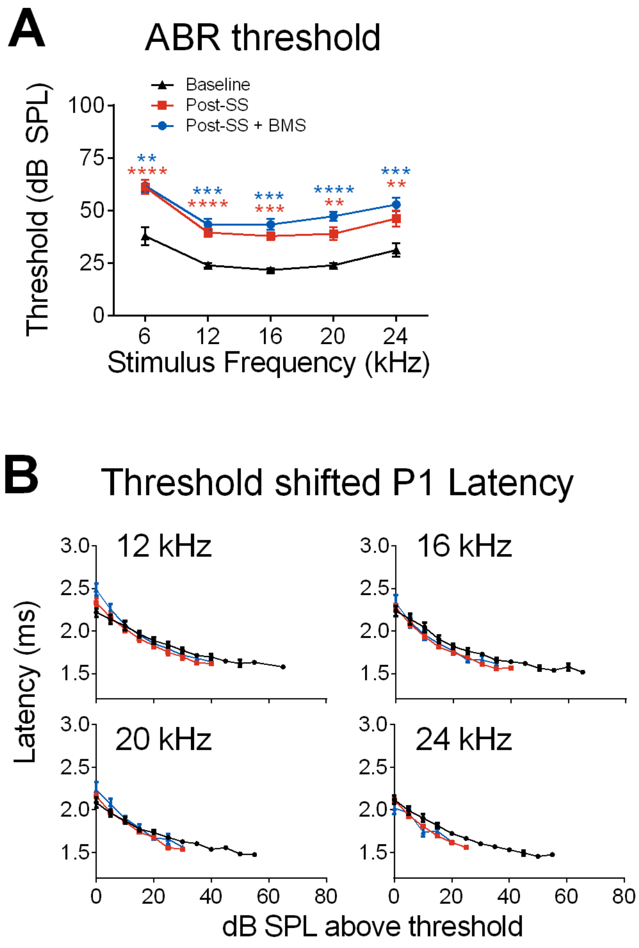

Supplemental Figure 2. **BMS-191011 treatment does not alter the effect of SS administration on ABR thresholds. (A)** A plot of ABR thresholds as a function of frequency. A two-way RM ANOVA of ABR thresholds showed a main effect of treatment ($F(1.233, 9.866) = 38.98$; $p<0.0001$) and frequency ($F(1.681, 13.45) = 52.26$; $p<0.0001$). Post-hoc analyses showed that thresholds increased relative to baseline following SS treatment, but were not further altered by BMS-191011 treatment. Orange and blue asterisks indicate a significant difference from baseline for the SS and SS+BMS-191011 groups, respectively. **(B)** Plots show the quantification of P1 latencies for 12, 16, 20 and 24 kHz tones presented at varying intensities. Stimulus intensity is expressed relative to the threshold intensity so that 0 dB SPL represents threshold for each animal in each condition (baseline, SS, SS+BMS-191011). Two-way ANOVAs indicated main effects of intensity (12 kHz: $F(5, 154) = 52.49$; $p<0.0001$; 16 kHz: $F(5, 152) = 49.99$; $p<0.0001$; 20 kHz: $F(5, 143) = 53.87$; $p<0.0001$; 24 kHz: $F(5, 102) = 5.870$; $p<0.005$), but not treatment (12 kHz: $F(2, 154) = 2.557$; 16 kHz: $F(2, 152) = 2.515$; 20 kHz: $F(2, 143) = 1.659$; 24 kHz: $F(2, 102) = 5.870$). N=8-12 per group, *$p<0.05$, **$p<0.01$, ***$p<0.001$, ****$p<0.0001$.

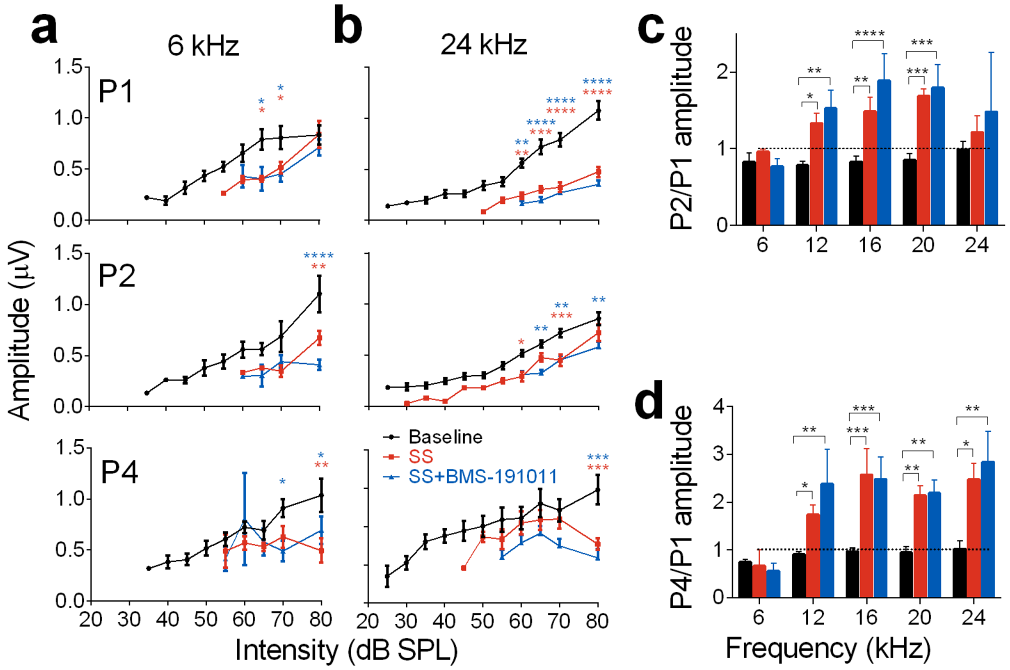

Supplemental Figure 3. **BMS-191011 treatment does not suppress the increased gain in early auditory structures caused by SS treatment. (A)** Plots show the quantification of P1, P2, and P4 amplitudes as a function of stimulus intensity for 6 kHz tones. Two-way ANOVAs indicated a significant effect of intensity for P1 and P2, but not P4 (P1: $F(3, 60) = 4.795$, $p< 0.005$; P2: $F(3, 55) = 4.902$, $p<0.005$; P4: $F(3, 62) = 0.664$). There is also a main effect of treatment (P1: $F(2, 60) = 7.864$, $p< 0.001$; P2: $F(2, 55) = 10.52$, $p<0.0001$; P4: $F(2, 62) = 5.446$, $p<0.01$). Post-hoc analyses indicated that when SS was administered alone or with BMS-191011, P1-P4 amplitudes were reduced relative to baseline at multiple intensities (orange and blue asterisks, respectively). BMS-191011 treatment did not significantly alter the effect of SS treatment. **(B)** Plots show the quantification of P1, P2, and P4 amplitudes as a function of stimulus intensity for 24 kHz tones. As for 6 kHz tones, two-way ANOVAs indicated a main effect of intensity for P1 and P2, but not P4 (P1: $F(3, 71) = 9.45$, $p< 0.0001$; P2: $F(3, 94) = 18.19$, $p<0.0001$; P4: $F(3, 102) = 0.622$). There was also a main effect of treatment (P1: $F(2, 71) = 73.55$, $p< 0.0001$; P2: $F(2, 94) = 22.47$, $p<0.0001$; P4: $F(2, 102) = 12.30$, $p< 0.0001$). Post-hoc analyses indicated that when SS was administered alone or with BMS-191011, P1-P4 amplitudes were reduced relative to baseline at multiple intensities (orange and blue asterisks, respectively). BMS-191011 treatment did not significantly alter the effect of SS treatment. **(C)** Graph of the amplitude of P2 relative to P1 for 60 dB tones show the effect of treatment on gain in the auditory brainstem both near the behaviorally-probed tinnitus frequency region (12-20 kHz) and farther from the tinnitus frequency region (6 and 24 kHz). Two-way ANOVA showed a main effect of frequency ($F(4, 111) = 3.04$; $p<0.05$) and treatment ($F(2, 111) = 15.85$; $p<0.0001$). Post-hoc analysis indicated that SS administration (orange) significantly increased the P2/P1 ratio relative to baseline (black) near the tinnitus frequency region, and that this was unaltered by co-treatment with BMS-191011 (blue). In contrast, SS administration did not significantly alter P2/P1 ratios at frequencies farther from the tinnitus frequency region. **(D)** Graph of the amplitude of P4 relative to P1 for 60 dB tones. Similar to the P2/P1 ratios, there was also a main effect of frequency ($F(4, 112) = 5.61$; $p<0.0005$)

and treatment ($F(2, 112) = 18.00$; $p<0.0001$). Post-hoc analyses indicated that SS administration (orange) significantly increased the P4/P1 ratio relative to baseline (black) for 12-24 kHz tones, and that this was unaltered by BMS-191011 treatment (blue). SS administration did not alter the P4/P1 ratio at 6 kHz. Dotted lines on the P2/P1 and P4/P1 graphs demarcate equal amplitudes (ratio = 1). N=8-12 per group, *$p<0.05$, **$p<0.01$, ***$p<0.001$, ****$p<0.0001$.

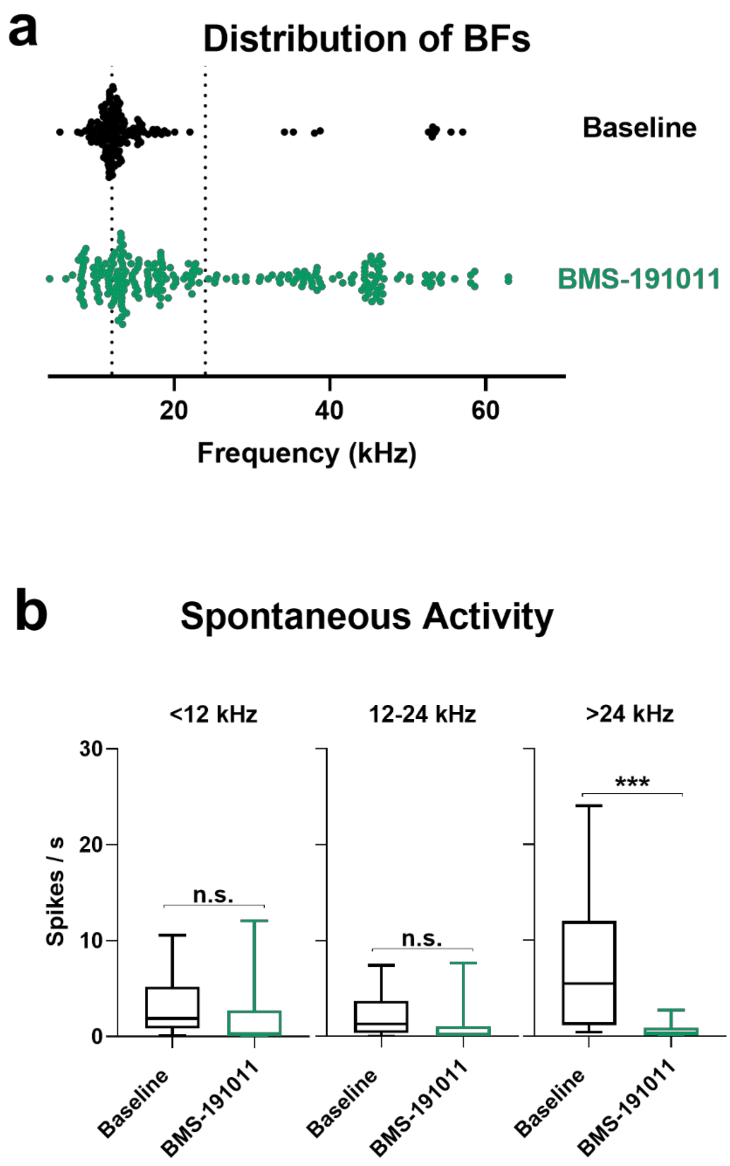

Supplemental Figure 4. **Local administration of BMS-191011 alone modifies spontaneous activity for high-frequency, but not mid- or low-frequency midbrain neurons. (A)** Total excitatory receptive fields (eFRAs) were recorded at baseline (black) and again 2 hours following local BMS-191011 administration over the IC (green) to determine best frequencies (BFs). Dotted lines indicate 12 and 24 kHz. Representation of high frequency BFs increases following BMS-191011 administration. **(B)** Plot of mean spontaneous activity rates measured at baseline (black), and again after BMS-191011 administration (green). A *t*-test showed a decrease in spontaneous activity for high-frequency neurons ($t(105)=3.627$; $p=0.0004$), but no effect of treatment for mid- ($t(174)=0.7912$; n.s.) and low-frequency ($t(113)=1.319$; n.s.) neurons.
